# Dual-functioning Targeted ADAM17 Blocker CD16 (TAB16) mediates selective ADAM17 inhibition in NK cells and engages overexpressed ADAM17 in tumor cells to induce cytotoxicity

**DOI:** 10.3389/fimmu.2026.1714022

**Published:** 2026-01-30

**Authors:** Kate J. Dixon, Robert Hullsiek, Sam Wang, Ryan R. Friess, Anders W. Matson, Yvette Soignier, Alexander J. Lenvik, Jianming Wu, Geoffrey T. Hart, Martin Felices, Bruce Walcheck

**Affiliations:** 1 Department of Veterinary and Biomedical Sciences, University of Minnesota, St. Paul, MN, United States; 2 Masonic Cancer Center, University of Minnesota, Minneapolis, MN, United States; 3 Department of Medicine, University of Minnesota, Minneapolis, MN, United States; 4 Center for Immunology, University of Minnesota, Minneapolis, MN, United States; 5 Stem Cell Institute, University of Minnesota, Minneapolis, MN, United States

**Keywords:** ADAM17 (a disintegrin and metalloprotease 17), ADCC - antibody-dependent cellular cytotoxicity, immunotherapy, natural killer (NK) cell, ovarian cancer

## Abstract

**Introduction:**

Natural killer (NK) cells are innate lymphocytes that kill tumor cells by natural cytotoxicity and antibody-dependent cell-mediated cytotoxicity (ADCC). Human NK cells mediate the latter process exclusively by the IgG Fc receptor CD16 (FcγRIIIA). Cell surface levels of this activating receptor are tightly regulated by the metalloprotease ADAM17, which cleaves CD16 upon NK cell activation or cellular stress. We have reported that Medi-1, a fully human IgG1 mAb, blocks ADAM17, and its Fc region is simultaneously engaged by CD16, inducing and prolonging its signaling, which synergizes with cytokine stimulation, such as IL-15. To exploit these distinctive features of Medi-1 while also addressing limitations of the mAb, such as the varied affinity by which CD16 binds to it due to receptor polymorphisms and the risk of broadly blocking ADAM17 activity, we engineered Targeted ADAM17 Blocker CD16 (TAB16).

**Methods:**

TAB16 was generated with a camelid heavy-chain variable domain specific to CD16 linked to a single-chain variable fragment derived from Medi-1. TAB16 was further modified by the linkage of an IL-15 moiety to generate TAB16/15. Primary human NK cells were treated with TAB16 or TAB16/15 and evaluated for proliferation by cell dilution dye, ADAM17 blocking, activation marker expression, and cytotoxicity against ovarian cancer cell lines in real-time by IncuCyte assays.

**Results:**

The TAB16 bispecific engager targeted NK cells and blocked ADAM17. A novel feature of TAB16 is its dual functionality, as it synergizes with IL-15 to enhance NK cell activation and proliferation and targets ADAM17 overexpressed on cancer cells to induce ADCC. TAB16 is a modifiable backbone to which additional functional components can be added, such as IL-15 (TAB16/15) for consolidated and multifaceted activity.

**Discussion:**

Our ADAM17-engaging platform offers a unique approach for targeted ADAM17 inhibition to augment the anti-tumor function of endogenous and therapeutic NK cells.

## Graphical Abstract

**Figure:**
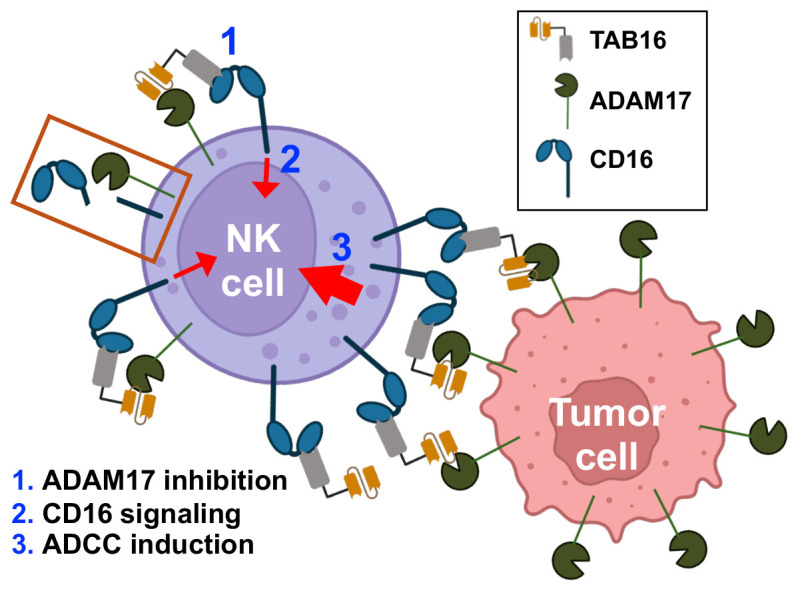

## Introduction

Natural killer (NK) cells are innate lymphocytes that interrogate cells of the body and can respond to neoplastic-transformed cells by releasing cytolytic factors and proinflammatory cytokines (1–3). NK cells mediate target cell killing through natural cytotoxicity and antibody-dependent cell-mediated cytotoxicity (ADCC) (1, 2, 4). The latter process can be facilitated by antibody therapies, allowing NK cells to target multiple tumor antigens, thereby reducing the risk of antigen escape (5, 6).

Mature circulating NK cells in humans express CD56, (CD56^dim^), CD16, and lack CD3. These cells exclusively mediate ADCC via CD16, an IgG Fc receptor (FcγRIIIA) (6–9). CD16 engagement can also direct NK cells to become memory-like, and when combined with cytokine activation, results in a population of NK cells with enhanced proliferation and anti-tumor cytotoxicity (10). CD16 is regulated differently than other activation receptors on NK cells in that it is rapidly cleaved from the cell membrane upon cell activation (11). This is mediated by a disintegrin and metalloproteinase 17 (ADAM17) (11–14), a proteolytic checkpoint induced by CD16 signaling and other stimuli that regulates cell-surface levels of CD16 and cell-cell contact (15, 16). ADAM17 activity is also increased by cell stress, such as hypoxia (17, 18), and NK cells downregulate CD16 in the tumor microenvironment (19–21), likely limiting the efficacy of NK cell ADCC. ADAM17 is broadly expressed, with activity being described in most cell lineages (22). Further, it is overexpressed in numerous malignancies, including colon cancer, ovarian cancer, lung cancer, breast cancer, pancreatic cancer, and prostate cancer, which is associated with a poor prognosis (23–29).

Our recent studies have demonstrated that the ADAM17 function-blocking monoclonal antibody (mAb) Medi-1, a fully human IgG1 antibody, when attached to NK cells, blocks the shedding of CD16 while being engaged by this FcR. This induces and prolongs CD16 signaling, which synergizes with cytokine stimulation, including IL-15 and IL-2, enhancing NK cell activation and proliferation *in vitro* and *in vivo* (30, 31). Established mechanisms underlying the effects of Medi-1 on cytokine-driven NK cell proliferation involve an enhanced upregulation of the co-stimulatory receptor CD137 (4-1BB), which engages CD137L on leukocytes such as monocytes (30), and inhibition of shedding of the ADAM17 substrate CD62L (L-selectin), enhancing NK cell trafficking to proliferation niches (31).

A potential limitation of using Medi-1, however, is that the mAb blocks ADAM17 in all cell types and is not NK cell-specific. The necessity of functional ADAM17 is demonstrated in murine models, as global knockout mice are embryonic lethal (32). In humans, loss-of-function mutations in ADAM17 result in severe dysfunction in multiple organs (33). An additional limitation of Medi-1 is that humans express polymorphic variants of CD16, including a variation at amino acid position 158, characterized by either phenylalanine (158F) or valine (158V), which affects its binding affinity to the Fc region of IgG (34–36). Most of the population expresses the 158F variant, which has a lower affinity for IgG than the 158V variant (37–40). This potential disparity in CD16 engagement of Medi-1, coupled with on-target off-NK cell inhibition of ADAM17, underscores the advantages of a more targeted approach to ADAM17 inhibition.

We developed a novel bispecific engager termed Targeted ADAM17 Blocker CD16 (TAB16), consisting of a single-chain variable fragment (scFv) derived from Medi-1 to block ADAM17 function linked to a camelid variable domain of a heavy chain-only antibody (VHH) that binds CD16 (cam16) with high affinity in a uniform manner (30). TAB16 directed ADAM17 inhibition to NK cells and augmented IL-15-driven activation and proliferation. Importantly, TAB16 also linked NK cells to ADAM17 overexpressed by ovarian cancer cell lines to mediate ADCC. The TAB16 platform is also amenable to modifications, such as the linkage of an IL-15 moiety, which we incorporated into the complex to generate TAB16/15, removing the variability of individual components. We show that TAB16/15 maintains NK cell specificity in blocking ADAM17 and increased proliferation while mediating ADCC against ovarian cancer cell lines with no additional IL-15 required. Our ADAM17-targeting strategy offers a novel means to prevent the shedding of critical receptors in a cell-selective manner.

## Methods

### Generation of TAB16 and TAB16/15

The anti-human ADAM17 mAb MEDI3622 has been previously described (30, 31). We have generated various Fc variants, including the human isotype IgG1 and an scFv from its nucleotide sequence, which we refer to as Medi-1 and Medi-scFv, respectively (30). Mammalian expression plasmids were generated by Gibson assembly of the TAB16 or TAB 16/15 sequence into the pMC.EF1α-MCS-SV40polyA parental minicircle cloning vector (System Biosciences, Palo Alto, California, USA, Cat. No. MN502A-1). Double-stranded DNA fragments (gBlocks) encoding the full-length fusion protein with an N-terminal signal peptide for secretion were synthesized by Integrated DNA Technologies (USA) and inserted into the plasmid backbone using the Gibson Assembly HiFi Kit (New England Biolabs, Ipswich, Massachusetts, USA, Cat. No. E5510S) according to the manufacturer’s instructions. *E. coli* DH5-α transformants were selected on kanamycin, verified by Sanger sequencing (University of Minnesota Genomics Center, Minneapolis, MN, USA), expanded, and plasmid DNA was purified using the ZymoPURE Maxiprep Kit (Zymo Research, Irving, California, USA. Cat. No D4202).

Plasmids were transfected into Expi293F cells (Gibco, Cat. No. A14527) using the Expi293 Expression System (Thermo Fisher Scientific, Waltham, Massachusetts, USA, Cat. No. A14635) according to the manufacturer’s protocol. Recombinant proteins were purified by immobilized metal affinity chromatography on an ÄKTA avant 150 system using HisTrap Excel columns (Cytiva, Marlborough, Massachusetts, USA, Cat. No. 17371206), utilizing the C-terminal 10× his tag for purification and detection. Desalting was performed with HiPrep 26/10 Desalting columns (Cytiva, Cat. No. 17508702). Protein purity and size were assessed by SDS–PAGE followed by One-Step Blue staining (Biotium, Fremont, California, USA, Cat. No. 21003–1 L) (**Supplementary Figure 1**). Concentrations were determined using the Qubit Protein Assay Kit (Invitrogen, Carlsbad, California, USA, Cat. No. Q33211).

### Flow cytometric analyses

NK cell phenotypic analyses were performed as previously described (30, 31). Briefly, fluorescence minus one (FMO) and appropriate isotype-matched antibodies were used for controls. A forward scatter-area/side scatter-area (FSC-A/SSC-A) plot was used to set an electronic gate on leukocyte populations, and an FSC-A/FSC-H plot was used to set an electronic gate on single cells, excluding doublets. All commercially available antibodies are listed in **Table 1**. Flow cytometry was performed in FACS buffer: sterile Dulbecco’s phosphate buffered saline (D-PBS) (Sigma-Aldrich, St. Louis, Missouri, USA Cat. No. D8537-100ML), 2% heat inactivated fetal bovine serum (HI FBS) (Gibco), and 0.1% sodium azide (Fisher Scientific, Waltham, Massachusetts, USA, Cat. No. S227I-100). For ADAM17 staining, SKOV-3 cells were labeled with 5 μg/ml of purified mouse anti-human TACE M220 (41) or an isotype-matched control antibody diluted in FACS buffer. The bound antibody was detected using 2.5 μg/ml goat anti-mouse IgG AF647. SKOV-3 cells were washed, then incubated for 20 minutes in 2% mouse serum (Thermo Fisher Scientific, Cat. No. 24-5544-94), washed, then directly labeled with 2.5 μg/ml of the following antibodies: mouse anti-human CD3 BV785, mouse anti-human CD56 PE/Cy7, and Tonbo Ghost Dye Red 710. All antibody incubations were performed at 4°C for 30 minutes, after which the cells were washed twice with FACS buffer and analyzed by flow cytometry. The cell viability dye Ghost Dye Red 710 (Tonbo Bioscience, San Diego, CA) was used to distinguish live vs. dead cells as per the manufacturer’s instructions. Phenotyping for CD25, CD69, and CD137 was done using similar conditions/incubations and gating strategy. Annexin V staining was performed per the manufacturer’s instructions (BioLegend, San Diego, California, Cat. No. 422201 and 640905). All samples were run immediately after staining, collecting at least 5,000 events within the NK cell gate (live/dead discrimination^−^, CD56^+^ CD3^−^) on a BD FACSCelesta Cell Analyzer (BD Bioscience, San Jose, CA) and analyzed using FlowJo v10.9.0 Software (BD Bioscience).

**Table 1:** Commercial antibodies.

Antibody	Fluorchrome conjugate	Antigen/ligand recognized	Source, catalog #
CD56	PE/Cy7	CD56	Biolegend, 318318
CD3	BV785	CD3	BioLegend, 300472
CD16	BV510	CD16	BioLegend, 302048
CD107a	APC	CD107a	Biolegend, 328620
CD69	PerCP/Cy5.5	CD69	BioLegend, 310927
CD25	FITC	CD25	BioLegend, 302604
CD62L	APC	CD62L	BioLegend 304810
CD137	APC	4-1BB	BioLegend, 309810
B7-H3	PE	B7-H3	BioLegend, 351004
IFNg	BV650	IFNg	BioLegend, 502537
Ghost Dye-Red 710	Red 710		Tonbo Biosciences, 13-0871
Trasturumab		HER2	Genentech
Daratumumab		CD38	Janssen Biotech, Inc
Anti-His	APC	His Tag	BioLegend 362605
F(ab')2 Fragment	APC		Jackson InmunoResearch 115-136-072
Isotype	AlexaFuor647		ThermoFisher A-21235
Isotype	APC		Biolegend 400120
Isotype	FITC		Biolegend 400110
Isotype	PerCP/Cy5.5		Biolegend 400147
Isotype	BV650		Biolegend 502537
Isotype	BV510		Biolegend 400171
Isotype	PE		Biolegend 400113

### Cell isolation and culture

PBMCs were obtained from healthy consenting adults at the University of Minnesota (IRB protocol # 9708M00134) in sodium heparin tubes (BD Bioscience, Cat No. 367874). PBMCs were isolated using Lymphocyte Separation Medium (Promocell, Heidelberg, Germany, Cat. No. C-44010). NK cells were enriched using a negative selection human NK Cell Isolation Kit (Miltenyi Biotec, Bergisch, Germany, Cat. No. 130-092-657) or (StemCell Technologies, Vancouver, BC, Canada, Cat. No. 19055). Isolated NK cells were ≥90% pure, as determined by CD56^+^ CD3^−^ staining and flow cytometry. Whole leukocyte isolation was performed by ammonium-chloride-potassium (ACK) lysis buffer (Gibco, Cat. No. A1049201) utilizing a ratio of 20% whole blood with 80% ice cold lysis buffer. Blood was inverted several times and incubated on ice for 5 minutes, spun at 300g for 5 minutes, and supernatant was discarded. ACK lysis was repeated if necessary. Leukocytes were resuspended in cold PBS and incubated with TAB16 for 30 minutes at 4°C. Leukocytes were activated or stained as described. PBMCs or enriched NK cells, either unlabeled or labeled with CellTrace Violet (CTV) Cell Proliferation Dye (Thermo Fisher Scientific, Cat. No. C34557) as per the manufacturer’s instructions, were cultured for 7 days at 37°C in 5% CO_2_ in RPMI 1640 media (Gibco) supplemented with 10% HI FBS and 1x antibiotic-antimycotic (anti-anti) (Gibco, Cat. No 15240062), in the presence or absence of IL-15 (Biological Resources Branch, National Cancer Institute). NK cell proliferation was analyzed via flow cytometry.

SKOV-3 cells (HTB-77), an ovarian adenocarcinoma cell line, and NK-92 (CRL-2407) cells were purchased from ATCC (Manassas, VA, USA). SKOV-3 cells stably expressing NucLightRed (NLR) were generated as previously described (42). Cells were maintained in McCoy’s 5A media (Gibco) supplemented with 10% HI FBS and 1x anti-anti. OVCAR8 and PA-1 cells were obtained from the laboratories of Drs. Melissa Geller and Martin Felices at the University of Minnesota and maintained in RPMI-1640 media supplemented with 10% HI FBS and 1x anti-anti. All cell lines were routinely tested for mycoplasma with the MycoAlert Mycoplasma test kit (Lonza, Basel, Switzerland, Cat. No. LT07-118).

### PMA/ionomycin activation

Whole leukocytes or lymphocytes were stimulated with phorbol 12-myristate 13-acetate (PMA) as described previously (11, 30). Briefly, leukocytes (3×10^5^/mL) were treated with or without 10 ng/ml PMA (Sigma Aldrich, Cat No. P8139) and 1 µg/mL ionomycin (AdipoGen Life Sciences, San Diego, California, USA, Cat. No. AG-CN2-0416-M001) for 30 minutes at 37°C in the presence or absence of Medi-1, TAB16, or an isotype-matched negative control antibody, at the indicated concentrations. The cells were then washed twice FACs buffer, treated with cell viability dye, and stained for CD16 and CD62L.

### IL-15 bioavailability assay

To establish the functional equivalence of IL-15 and TAB16/15, a bioassay was performed using IL-15-responsive NK-92 cells, as we have previously described (43–45). Briefly, NK-92 cells were cultured in IL-2-free media (RPMI supplemented with 10% FBS, 1× anti-anti) for 24 hours, then plated in a clear-bottom 96-well plate (Corning, Corning, NY, USA, Cat. No. 3603) with IL-15 or TAB16/15 serially diluted 1:3 eight times, starting from 120 nM. After 48 hours, resazurin (R&D Systems, Minneapolis, Minnesota, USA, Cat. No. AR002) was added to the samples, and fluorescence readings were measured using a Clariostar plate reader (BMG Labtech, Cary, North Carolina, USA) after 3 hours. Dose-response curves were generated to determine the EC50 values for the respective versions of IL-15. From the average of three technical replicates, the IL-15 moiety in the TAB16/15 was determined to be 10.72 times less potent than NCI IL-15. Hence, the “equifunctional” dose of TAB16/15 for IL-15 at 10 ng/ml (0.769 nM) would be 8.25 nM, as those amounts elicited equivalent NK-92 stimulation.

### Tumor cell cytotoxicity assays

To measure cell cytotoxicity in real-time, NLR tumor targets (SKOV-3, OVCAR8, PA-1) cells were plated at a density of 4x10^3^ cells/well on a 96-well flat bottom tissue culture treated plate. Target cells were incubated for 4 hours at 37°C in 5% CO_2_ in RPMI 1640 media (Gibco) supplemented with 10% HI FBS nd 1x anti-anti (Gibco) to allow for adherence prior to target cell addition. Enriched NK cells were added at the indicated E:T ratios in RPMI 1640 media with 10% heat inactivated FBS and 1x anti-anti at 37°C and 5% CO2. Fluorescent images of live cells were obtained hourly for the duration of the assay using an IncuCyte SX3 live cell imaging and analysis system (Sartorius, Gottingen, Germany), as we have previously described (30, 42, 46). Data are presented as double-normalized frequency of target cells remaining. CD107a and IFN-γ staining was done as previously described (12, 30). Briefly enriched NK cells were isolated from healthy donors, co-cultured with SKOV-3 cells at an E:T of 1:1 for 5 hours. After 1 hour of co-culture, monensin (BD Bioscience, Cat. No. BDB554724) and Brefeldin A (BD Bioscience, Cat. No. 555029) was added for the remaining 4 hours. Enriched NK cells were washed and stained with antibodies and analyzed by flow cytometry. Degranulated NK cells were identified as CD3^-^, CD56^+^, CD107a^+^ after doublet discrimination and live/dead exclusion with Ghost Red Dye 710. CD16 cleavage during ADCC was measured following incubation of 1x10^5^ SKOV-3 cells with 1x10^5^ enriched NK cells, incubated at 37°C in 5% CO_2_ in RPMI 1640 media supplemented with 10% HI FBS and 1x anti-anti for 5 hours with the noted stimulations. Cells were washed and stained with antibody and analyzed for flow cytometry.

### CRISPR/Cas9 knockout of ADAM17 in SKOV-3 cells

Single guide RNAs (sgRNA) for human ADAM17 were designed using Synthego’s Knockout Guide Design tool (https://design.synthego.com/#/). Recombinant Cas9 protein was purchased from IDT (Coralville, Iowa, USA). CRISPR/Cas9 ribonucleoprotein complexes (RNPs) were assembled by mixing 100 pmol of sgRNA with 30 pmol of Cas9. After assembly, RNP complexes rested at room temperature for 15 minutes. RNPs were combined with the Amaxa nucleofector solution from the SF Cell Line 4D-Nucleofector X Kit (Lonza, Cat. No. V4XC-2032). SKOV-3 NLR cells (2×10^5^) were then resuspended in the RNP/nucleofector solution and transferred to a 20 µL Nucleocuvette strip and nucleofected with the Amaxa 4D Nucleofector (Lonza) program FE-132. Nucleofected SKOV-3 cells were gently resuspended with warm culture medium and transferred into a 24-well plate for initial expansion. SKOV-3 cells lacking ADAM17 expression were confirmed by flow cytometry.

### Statistical analysis

Data are presented as the mean ± SD. Compiled data are from at least three independent experiments using separate blood donors. Statistical analysis was conducted, and significance was determined using GraphPad Prism version 10.0.3 for IOS, GraphPad Software (La Jolla, CA). Comparison between two treatments was computed using the paired two-tailed Student’s t-test. Comparison among three or more treatments was made using one-way or two-way ANOVAs, as appropriate, followed by the indicated *post hoc* test. The data generated in this study are available upon request from the corresponding author.

## Results

### TAB16 generation and specificity to NK cells

**Figure 1:**
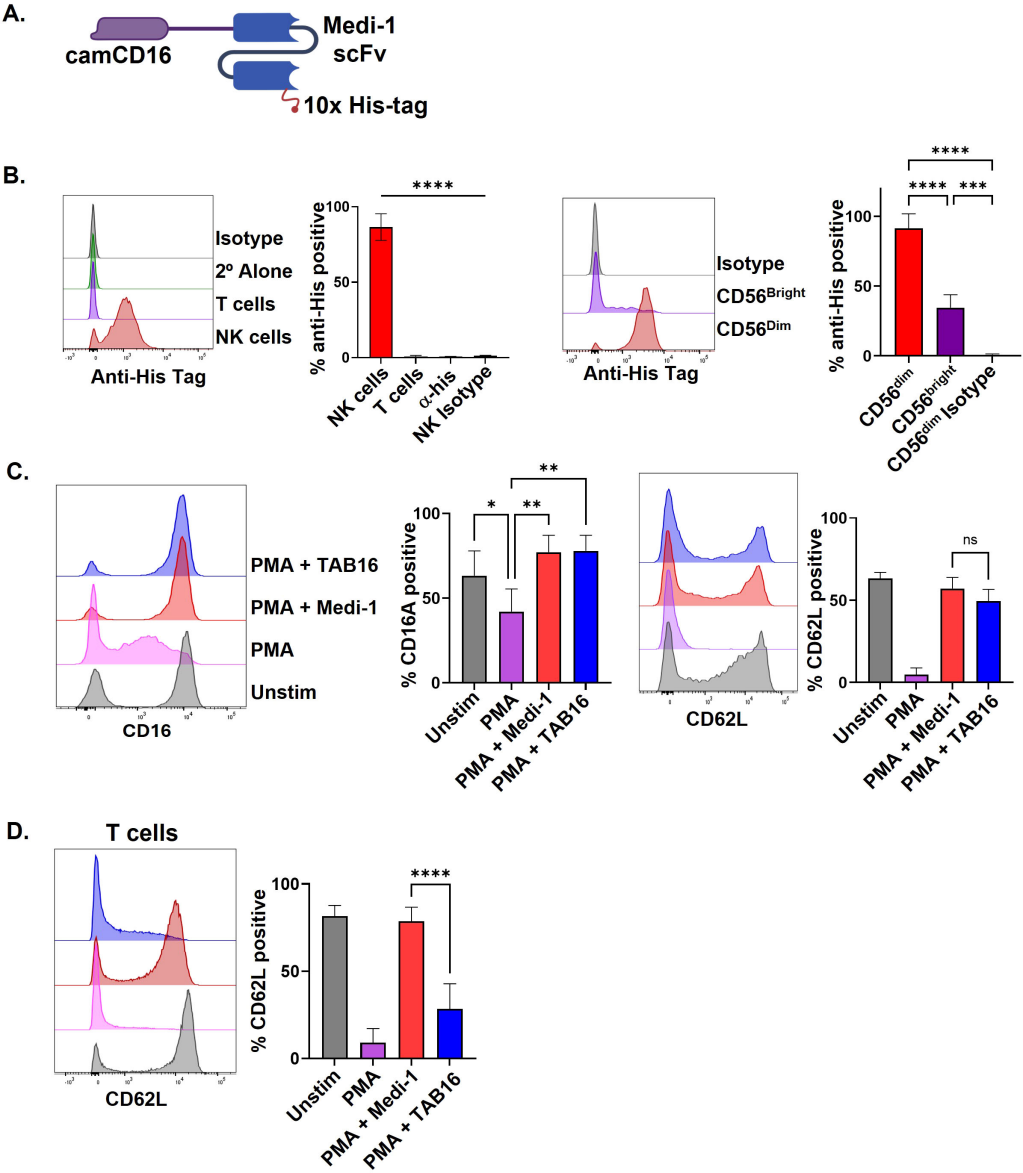
TAB16 selectively blocks ADAM17 in NK cells. **(A)** Schematic of TAB16 containing a C-terminus 10x his tag. **(B)** Flow cytometry of PBMCs cultured with 33 nM TAB16 for 30 minutes, thoroughly washed, and stained with an anti-his tag antibody along with antibodies to distinguish NK cells (CD56^+^ CD3^-^) and T cells (CD56^-^ CD3^+^). PBMCs treated with TAB16 were also stained with an isotype-matched negative control antibody (Isotype) and/or a goat anti-mouse IgG secondary antibody (2° Alone) to assess background staining levels. Y-axis = cell number. **(C, D)** PBMCs were activated with PMA (10 ng/ml) and ionomycin (1 μg/ml) for 1 hour at 37°C in the presence of Medi-1 (33 nM) or TAB16 (33 nM). NK cells (CD56^+^ CD3^-^) or T cells (CD56^-^ CD3^+^) were analyzed by flow cytometry for surface expression levels of CD16 and CD62L. Data shown as mean +/– SD, n=3–6 donors. *p<0.05; **p<0.01; ***p<0.001; ****p<0.0001; ns, not significant. Statistical significance was determined by one-way ANOVA with a Tukey *post hoc* test.

Medi-1 is a fully human IgG1 mAb specific to ADAM17 and completely blocks its function (30). When attached to NK cells, Medi-1 is engaged by CD16 and prevents its shedding, inducing and extending cellular activation (30). Given the numerous substrates and importance of ADAM17 in cell homeostasis (22), targeting ADAM17 inhibition to specific cells is likely to be important if prolonged inhibition is necessary. Thus, we generated a multi-engager antibody consisting of an scFv from Medi-1 (Medi-scFv) and a camelid VHH that binds CD16 (cam16). We refer to this bispecific engager as a Targeted ADAM17 Blocker CD16 (TAB16) (**Figure 1A**). The cloned product also contained a C-terminal 10x his tag. The TAB16 construct was transfected into Expi293 cells for mammalian expression and isolated by chromatography (30). Using the his tag present in TAB16, flow cytometry was performed to assess its binding to lymphocytes. T cells and NK cells both express ADAM17 (12); however, TAB16 specifically bound to NK cells and preferentially to the CD56^dim^ subset that primarily expresses CD16 (**Figure 1B**; **Supplementary Figure 2A**). These findings reveal that TAB16 selectively binds to cells expressing CD16.

CD16 and CD62L are well-established substrates of ADAM17 (15). We examined the effects of TAB16 on their expression following NK cell activation. PBMCs were stimulated with PMA and ionomycin, a well-characterized stimulant of ADAM17 activity (30), in the presence of TAB16 or Medi-1. Both prevented CD16 and CD62L downregulation upon NK cell activation (**Figure 1C**). T cells do not express CD16; however, CD62L underwent a rapid downregulation during PMA and ionomycin treatment (**Figure 1D**). This was blocked by Medi-1 but not TAB16 (**Figure 1D**). These findings demonstrate that TAB16 binds to NK cells, but not lymphocytes lacking CD16, to selectively neutralize ADAM17 activity. CD16 consists of two isoforms: FcγRIIIA on NK cells and FcγRIIIB on neutrophils, encoded by highly homologous genes (47). We observed that TAB16 also stained peripheral blood neutrophils and inhibited their downregulation of CD62L (**Supplementary Figures 2B, C**).

**Figure 2:**
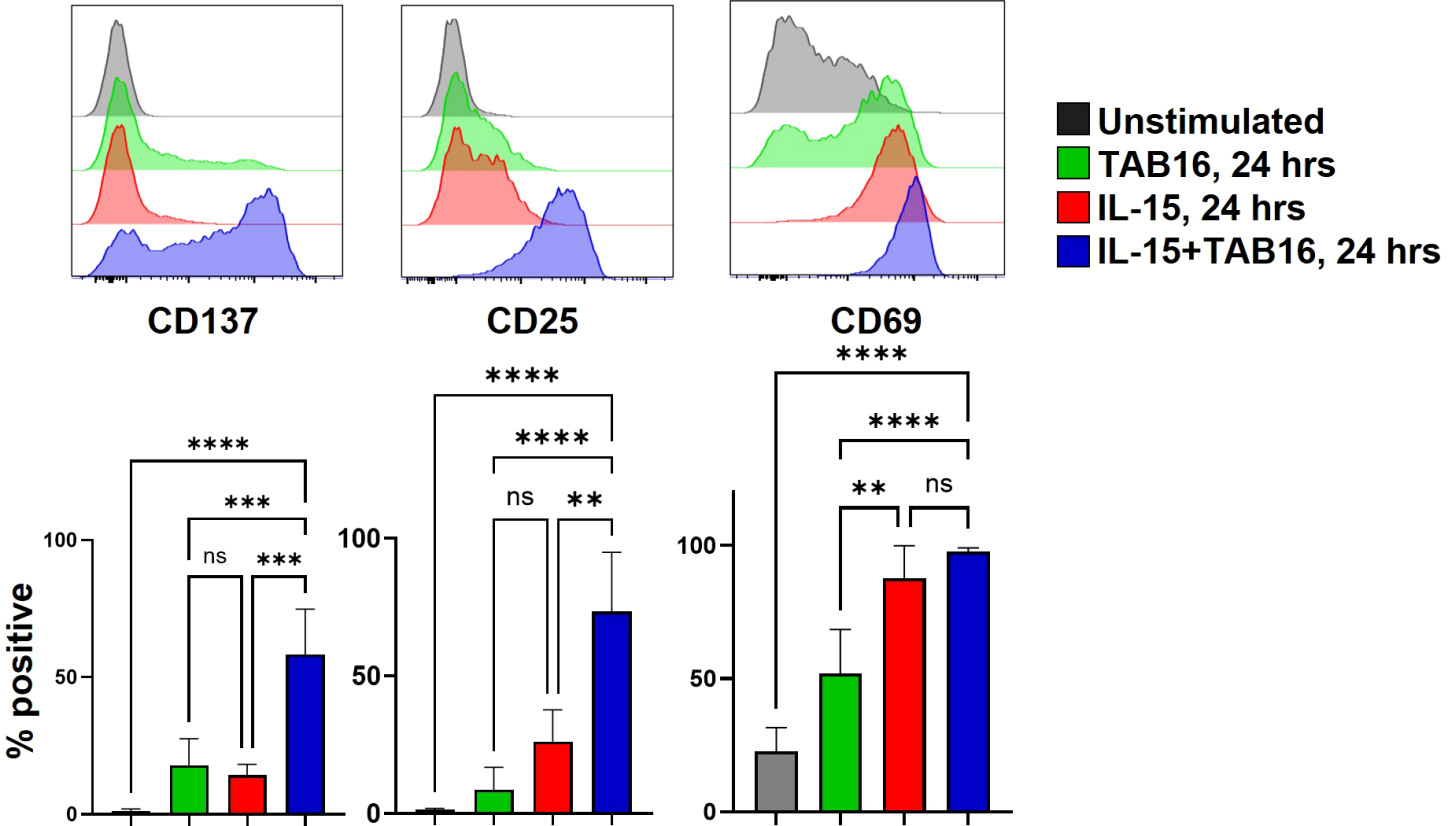
TAB16 enhances the activation of IL-15-stimulated NK cells. Enriched NK cells (>90%) were cultured with human Medi-1 (33nM), TAB16 (33 nM), +/– IL-15 (10 ng/ml) for 24 hours. NK cells (CD56^+^ CD3^–^) were analyzed for their expression levels of CD25, CD137, and CD69 by flow cytometry. Representative flow plots (top), y-axis = cell number. Cumulative data showing the percent with mean +/– SD. n=6 donors. **p<0.01; ***p<0.001; ****p<0.0001; ns, not significant. Statistical significance was determined by one-way ANOVA with a Tukey *post hoc* test.

We have demonstrated that Medi-1 acts in synergy with IL-15 to enhance the activation of NK cells, inducing a more rapid and robust upregulation of the early activation markers CD137 and CD25 (30). To determine if the same process occurs with TAB16, enriched NK cells were stimulated with IL-15 (10 ng/ml) with or without TAB16 for 24 hours. NK cells stimulated with IL-15 alone demonstrated a modest upregulation in expression of CD137 and CD25, which was markedly enhanced in the presence of TAB16 (**Figure 2**). CD69 upregulation is an early and very sensitive activation marker whose expression was greatly increased upon IL-15 stimulation and further augmented in the presence of TAB16 (**Figure 2**). Though the percentage of NK cells expressing CD69 was not different when stimulated with IL-15 + TAB16 (**Figure 2**), CD69 levels were found to be significantly higher on NK cells stimulated with IL-15 + TAB16 versus IL-15 alone (21.91 ± 4.529 vs. 10.43 ± 5.630, respectively, MFI fold change compared to day 0 ± SD, p=0.0187). We have previously shown that Medi-1, upon attachment to ADAM17 and engagement by CD16, induced ZAP-70 and SYK phosphorylation and NK cell stimulation (30). The treatment of NK cells with TAB16 alone induced a modest upregulation of CD25, CD69, and CD137 (**Figure 2**), indicating that its attachment to CD16 also resulted in low-level NK cell activation.

### TAB16 enhances IL-15-mediated NK cell proliferation

**Figure 3:**
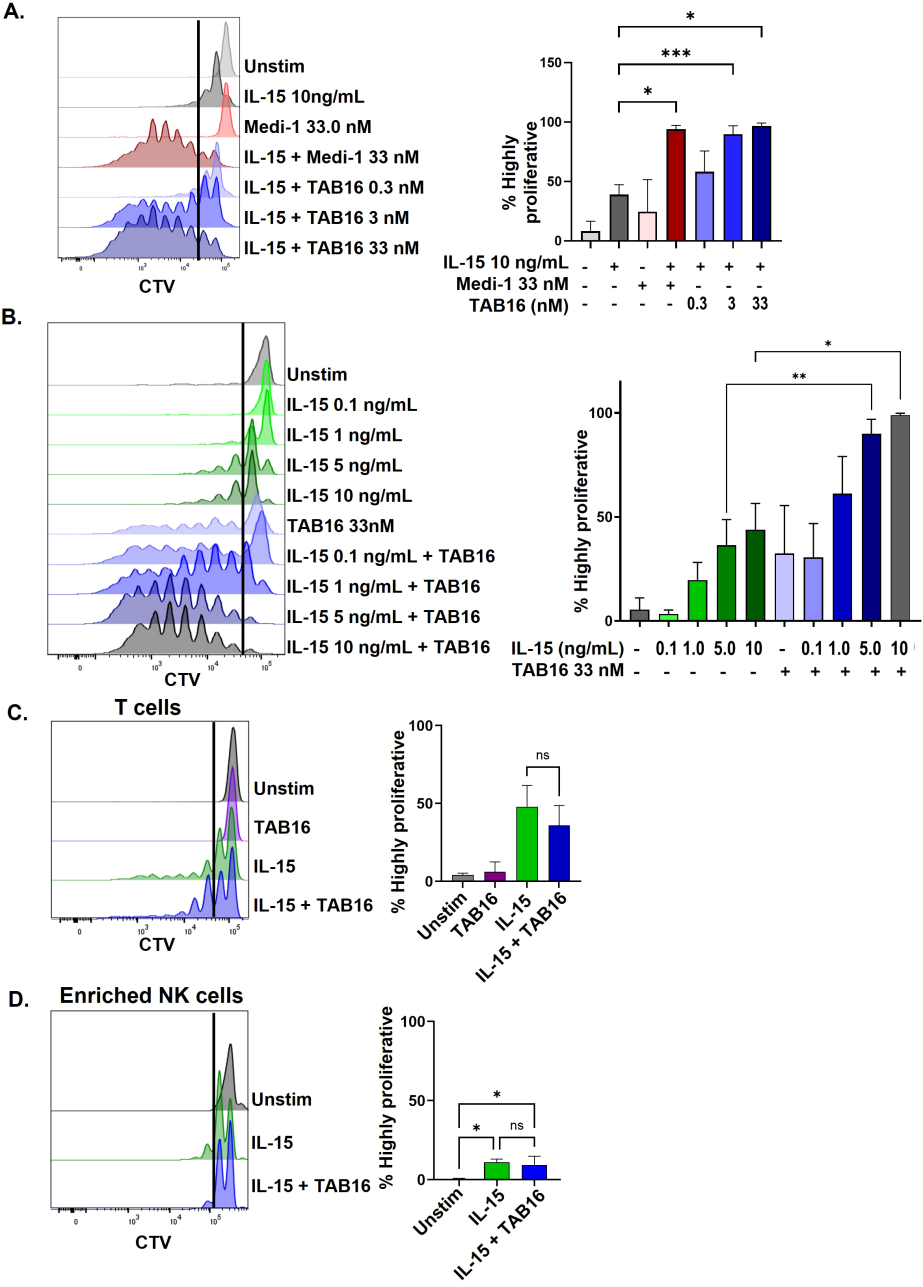
TAB16 enhances IL-15-mediated NK cell proliferation. **(A)** PBMCs were labeled with CTV to track cell division and cultured for 7 days +/- IL-15 (10 ng/ml) +/– TAB16 (0.3–33 nM). NK cells (CD56^+^ CD3^–^) were analyzed for CTV dilution by flow cytometry. Representative data are shown (left panel). Highly proliferative cells are indicated to the left of the line. The percentage of these cells for each condition was quantified (right panel). **(B)** PBMCs were labeled with CTV and cultured for 7 days +/– IL-15 (0.1–10 ng/ml) +/– TAB16 (33 nM), cells were analyzed as described in panel **(A, C)** CTV-labeled PBMCs were cultured with IL-15 (10 ng/ml) +/– TAB16 (33 nM), for 7 days, and then analyzed by flow cytometry gating on T cells (CD56^–^CD3^+^). **(D)** Purified NK cells (>90%) were treated as described in panel C and analyzed by flow cytometry. Mean +/– SD, n=3–5 donors. *p<0.05; **p<0.01; ***p<0.001; ns, not significant. Statistical significance was determined by one-way ANOVA with a Tukey *post hoc* test.

Our previous work established that Medi-1 enhanced IL-15-driven NK cell proliferation *in vitro* and *in vivo* (30, 31). This occurred in the presence of other PBMCs, such as monocytes, which transpresent IL-15 to NK cells and express CD137L for costimulation (30). To determine if TAB16 had a similar effect *in vitro*, PBMCs were labeled with CellTrace Violet (CTV) and cultured with IL-15 in the presence of either Medi-1 or TAB16. PBMCs were stimulated with 10 ng/ml of IL-15 for 7 days, a standard concentration and time frame used for lymphocyte expansion (19, 30, 31). TAB16 was examined at various concentrations (0.3, 3, and 33 nM). At concentrations ≥3 nM, TAB16 significantly enhanced NK cell proliferation compared to IL-15 alone (**Figure 3A**). To determine the effects of TAB16 on IL-15 responsiveness, PBMCs were labeled as described above and incubated with 33 nM of TAB16 with various concentrations of IL-15 (0.1, 1, 5, and 10 ng/ml). TAB16 was observed to significantly increase NK cell sensitivity to IL-15-mediated proliferation (**Figure 3B**). TAB16, however, did not enhance IL-15-mediated proliferation of T cells within the PBMC population (**Figure 3C**), further demonstrating its specificity for NK cells. In contrast to NK cells in the presence of other PBMCs, IL-15-driven proliferation of enriched NK cells was not increased by TAB16 (**Figure 3D**). Taken together, the above findings show that TAB16 selectively enhances IL-15-driven NK cell activation and proliferation across a range of IL-15 concentrations, similar to Medi-1 (30).

### TAB16 links NK cells to tumor cells overexpressing ADAM17 to induce ADCC

**Figure 4:**
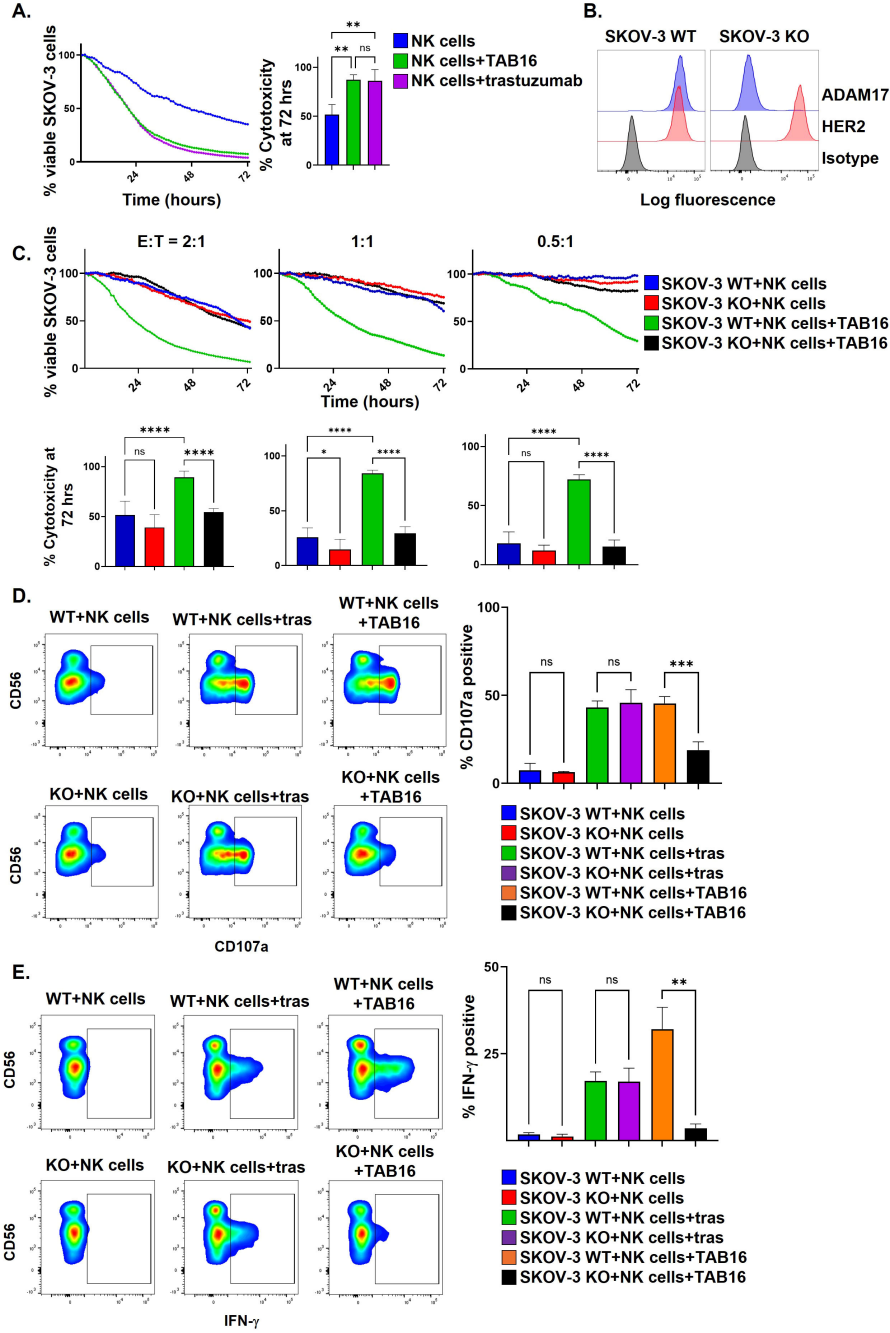
TAB16 links NK cells to ovarian tumor cells to induce ADCC. **(A)** Enriched NK cells were co-cultured with SKOV-3 NLR cells at an E:T ratio of 2:1 for 72 hours in the presence of IL-15 (10 ng/ml), +/- TAB16 (33 nM), +/- trastuzumab (5 μg/ml). Cytotoxicity was assessed by IncuCyte live cell imaging. The percentage of SKOV-3 NLR cells was double normalized to SKOV-3 NLR cells alone. The percent cytotoxicity at 72 hours was quantified. **(B)** SKOV-3 NLR WT and SKOV-3 NLR ADAM17 KO cells were analyzed for their ADAM17 and HER2 expression levels by flow cytometry. Representative histograms plots, y-axis = cell number. **(C)** Enriched NK cells (>95%) were cultured with SKOV-3 NLR WT or SKOV-3 NLR KO cells with IL-15 (10 ng/ml) +/– TAB16 (33 nM). The E:T ratio is indicated. Cytotoxicity was determined as described in panel **(A)** Representative IncuCyte plots (above), cumulative killing at 72 hours (graphed below). **(D)** Enriched NK cells were incubated with SKOV-3 NLR WT or SKOV-3 NLR KO cells +/– trastuzumab (tras) (5 μg/ml) +/- TAB16 (E:T of 1:1) for 4 hours. NK cells were stained for CD56, CD3, CD107a, and live/dead discrimination and examined by flow cytometry (live, CD56^+^ CD3^-^, CD107a^+^ cells were graphed). **(E)** Enriched NK cells were incubated with SKOV-3 NLR WT cells, IL-15 (10 ng/ml) +/– trastuzumab (tras) (5 μg/ml) or TAB16 (33 nM) at an E:T of 1:1 for 24 hours. NK cells were stained for CD56, CD3, IFN-γ, and live/dead discrimination and examined by flow cytometry (live, CD56^+^ CD3^-^, IFN-γ ^+^ cells were graphed). Representative flow plots (left panels) and cumulative data (right panel) are shown. Mean +/– SD, n=3–6 donors. *p<0.05; **p<0.01; ***p<0.001; ****p<0.0001; ns, not significant. Statistical significance was determined by one-way ANOVA with a Tukey *post hoc* test.

**Figure 5:**
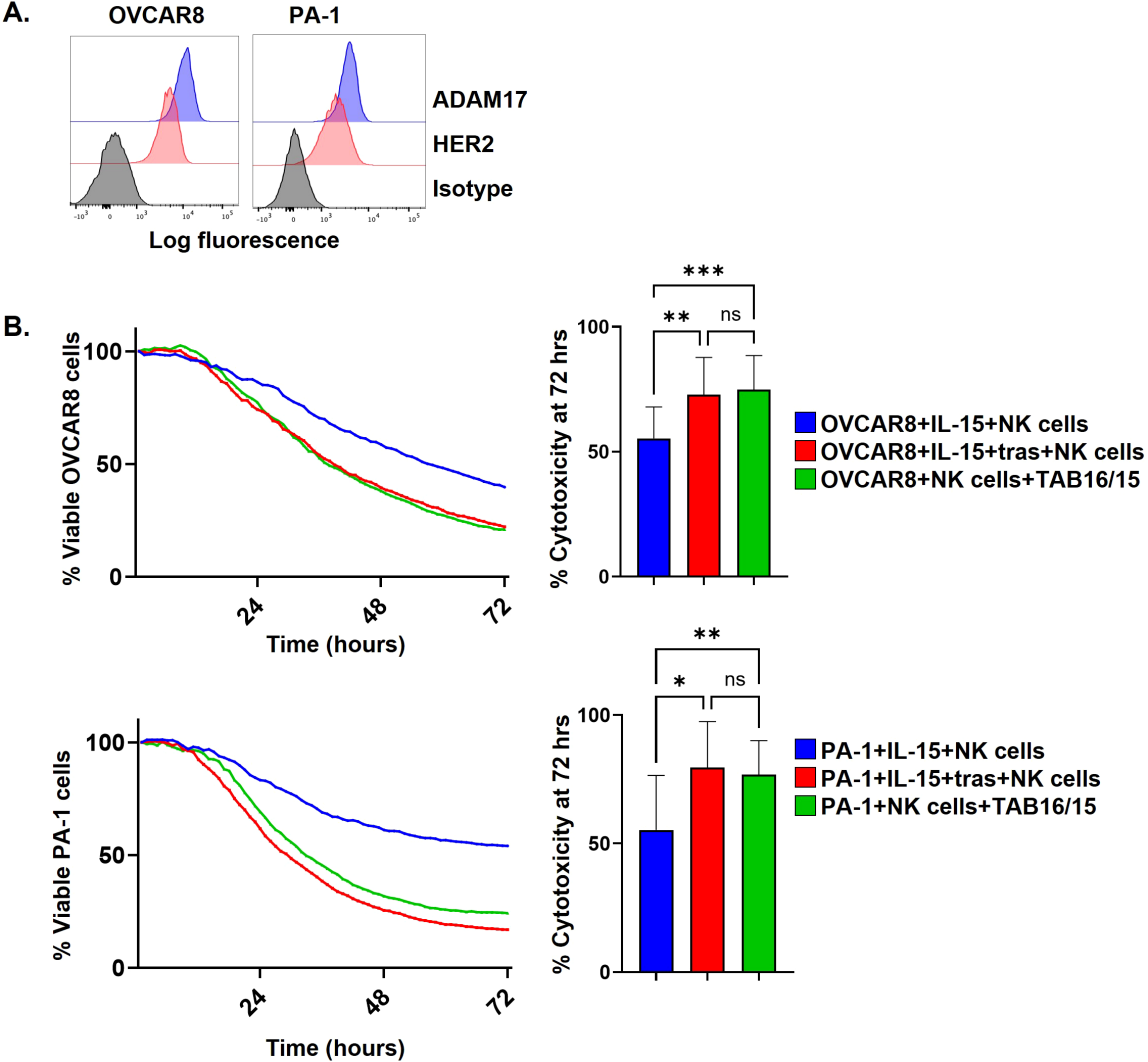
TAB16/15 mediated ADCC against ovarian tumor cell lines expressing ADAM17. **(A)** Human ovarian carcinoma cell line, OVCAR8 NLR, and human epithelial ovarian cancer cell line, PA-1 NLR were analyzed for their ADAM17 and HER2 expression levels by flow cytometry. Representative histograms plots y-axis = cell number. **(B)** Enriched NK cells were co-cultured with OVCAR8 NLR (top) or PA-1 NLR (bottom) cells at an E:T ratio of 2:1 for 72 hours in the presence of IL-15 (10 ng/ml) +/– trastuzumab (5 μg/ml) or TAB16/15 (8.25 nM). Cytotoxicity was assessed by IncuCyte live cell imaging. The percentage of OVCAR8-NLR or PA-1 NLR cells was double normalized to target cells alone. The percent cytotoxicity at 72 hours was quantified (right). Mean +/- SD, n = 6. *p<0.05; **p<0.01; ***p<0.001; ns, not significant. Statistical significance was determined by one-way ANOVA with a Tukey *post hoc* test.

Given TAB16’s synergy with IL-15 in promoting NK cell activation, we investigated its impact on NK cell anti-tumor activity. Cytotoxicity assays were performed with enriched NK cells to prevent the confounding effect from enhanced proliferation. NK cells were co-cultured with the ovarian cancer cell line SKOV-3 NLR at an E:T ratio of 2:1, in an IncuCyte real-time cytotoxicity imaging assay. NK cells and SKOV-3 NLR cells were co-cultured in 10 ng/ml of IL-15 ± TAB16 or the HER2 mAb trastuzumab. IL-15 with the addition of TAB16 or trastuzumab showed significantly improved killing by NK cells compared to IL-15 alone (**Figure 4A**). Of interest is that NK cells in the presence of TAB16 induced equivalent tumor cell killing as trastuzumab-induced ADCC (**Figure 4A**). It is well described that ADAM17 is overexpressed in several human cancers (48), including ovarian cancer (24, 29). We stained SKOV-3 cells (**Figure 4B**), as well as OVCAR8 and PA-1 (**Figure 5A**) for ADAM17 and found high surface expression by flow cytometry. It is possible that TAB16 could link CD16 on NK cells to ADAM17 on these ovarian cancer cell lines and induce ADCC. To test this, we used CRISPR/Cas9 genome editing to generate ADAM17 knockout (KO) SKOV-3 NLR cells. SKOV-3 KO NLR cells demonstrated essentially no ADAM17 expression but maintained HER2 surface levels (**Figure 4B**). Enriched NK cells were co-cultured with SKOV-3 wildtype (WT) or SKOV-3 KO cells in the presence of IL-15 +/- TAB16 at various E:T ratios. SKOV-3 cell lysis was assessed by IncuCyte monitoring. At all E:T ratios, TAB16 significantly increased tumor cell lysis of SKOV-3 WT cells but not against SKOV-3 KO cells (**Figure 4C**; **Supplementary Figure 3**). NK cells killed SKOV-3 WT and KO cells with similar efficiency in the absence of TAB16 (**Figure 4C**), demonstrating that ADAM17 gene deletion did not alter the susceptibility of SKOV-3 cells to NK cell natural cytotoxicity. To further evaluate the effects of TAB16 on NK cell function, enriched cells were co-cultured with SKOV-3 WT and SKOV-3 KO cells in the presence of IL-15 +/- TAB16 at an E:T ratio of 1:1, and then stained for surface levels of CD107a, an established marker of degranulation, and intracellular levels of IFN-γ. TAB16 significantly increased NK cell degranulation when co-cultured with SKOV-3 WT compared to SKOV-3 KO cells. Moreover, this occurred in the CD56^dim^ NK cell subset that expresses CD16 (**Figures 4D, E**). Further, NK cells were cultured under the same conditions and evaluated for proinflammatory IFN-γ generation. NK cells co-cultured with WT SKOV-3 cells and TAB16 showed increased IFN-γ generation compared to TAB16 stimulated NK cells co-cultured with ADAM17 KO SKOV-3 cells (**Figure 4E**). These data show that in addition to blocking ADAM17 activity in NK cells, TAB16 can link NK cells to ADAM17 overexpressed by tumor cells and induce ADCC, which is likely enhanced by inhibiting CD16 shedding upon NK cell activation during this process. Indeed, CD16 downregulation was induced by trastuzumab-mediated ADCC (**Supplementary Figure 4A**). Moreover, the linkage of NK cells to SKOV-3 cells by a bispecific engager that binds to CD16 and the tumor antigen B7-H3, referred to as B7H3 bispecific killer engager (BiKE) (45), also induced CD16 downregulation, whereas their linkage by TAB16 did not (**Supplementary Figure 4A**). B7-H3 is expressed on SKOV-3 cells (**Supplementary Figure 4B**) (49).

**Figure 6:**
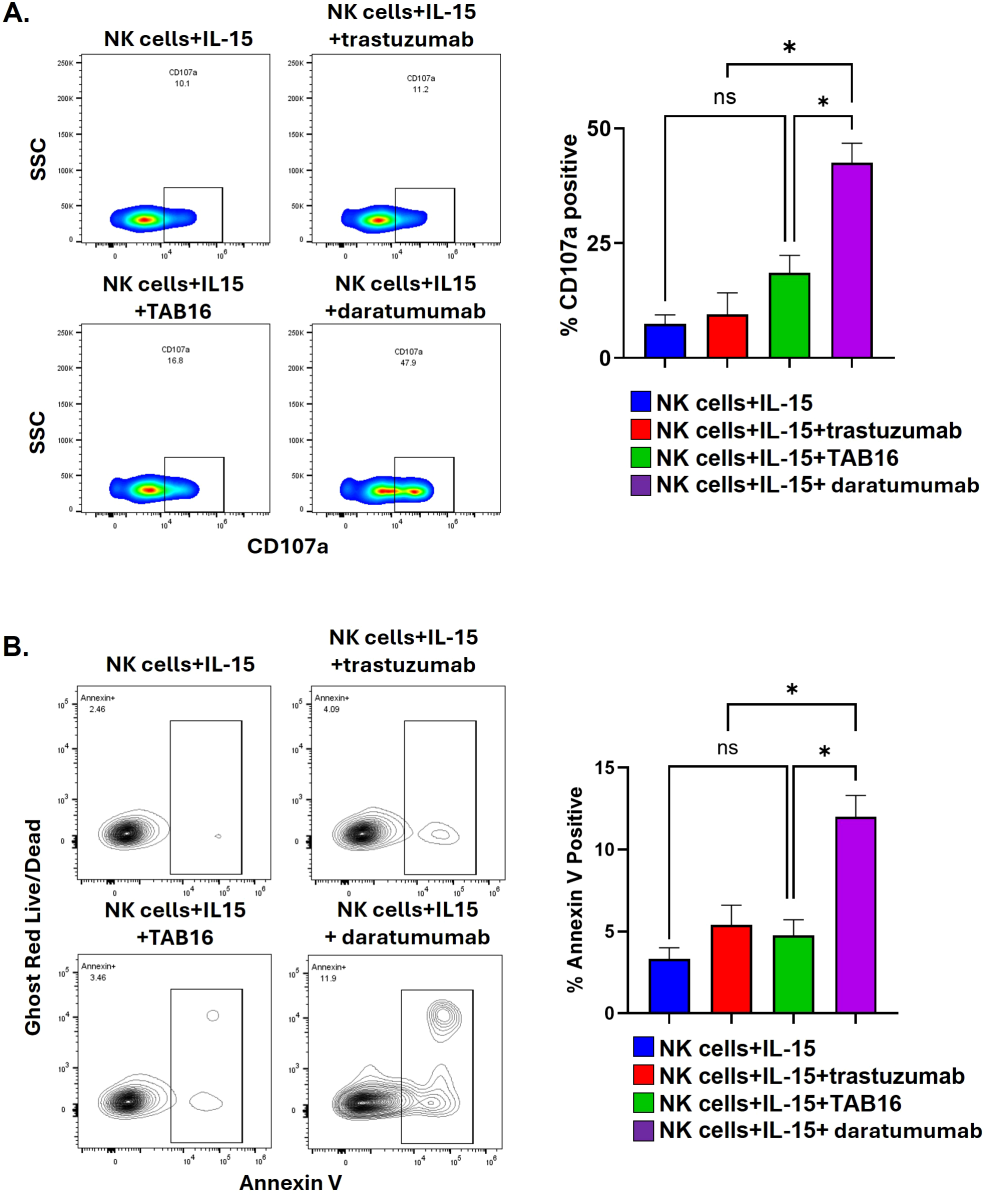
TAB16 does not induce NK cell fratricide. **(A)** Enriched NK cells (>90%) were incubated with IL-15 (10 ng/ml), +/- trastuzumab (5 μg/ml), TAB16 (33 nM), or daratumumab (5 μg/ml) for 5 hours. NK cells were stained for CD3, CD56, CD107a, and live/dead discrimination (live, CD56^+^ CD3^–^, CD107a^+^ cells graphed). Representative flow plots (left panels) and cumulative data (right panel). **(B)** Enriched NK cells (>95%) were incubated with IL-15 (10 ng/ml), +/- trastuzumab (5 μg/ml), TAB16 (33 nM), or daratumumab (5 μg/ml) for 24 hours. NK cells were stained for CD3, CD56, Annexin V, live/dead discrimination (CD56^+^ CD3^–^, Annexin V^+^ cells graphed). Representative flow plots (left panels) and cumulative data (right panel). Mean +/- SD, n = 3. *p<0.05; ns, not significant. Statistical significance was determined by one-way ANOVA with a Tukey *post hoc* test.

We previously established that CD16 engagement of Medi-1 attached to ADAM17 on NK cells did not result in NK cell degranulation and fratricide (30). Because the cam16 arm of TAB16 is likely to bind CD16 with higher affinity than CD16 engagement of Medi-1, we also examined whether TAB16 induced NK cell degranulation and fratricide. Enriched NK cells were incubated with IL-15 in combination with TAB16 or daratumumab, a fully human IgG1 antibody specific to CD38 (50). CD38 is expressed by NK cells and daratumumab is known to induce fratricide (50, 51). IL-15 plus trastuzumab (human IgG1), which does not bind to NK cells, was examined as a negative control for daratumumab. As expected, daratumumab induced a significant upregulation of CD107a by NK cells when compared to trastuzumab (**Figure 6A**). TAB16 induced a marginal but not significant increase in CD107a upregulation (**Figure 6A**). As expected, NK cells incubated with daratumumab had significantly increased Annexin V staining compared to TAB16 or trastuzumab (**Figure 6B**). Thus, these data show that TAB16, similar to Medi-1 (30), did not induce substantial NK cell fratricide.

### TAB16 complexed with IL-15 augments NK cell cytotoxicity without additional IL-15 support

**Figure 7:**
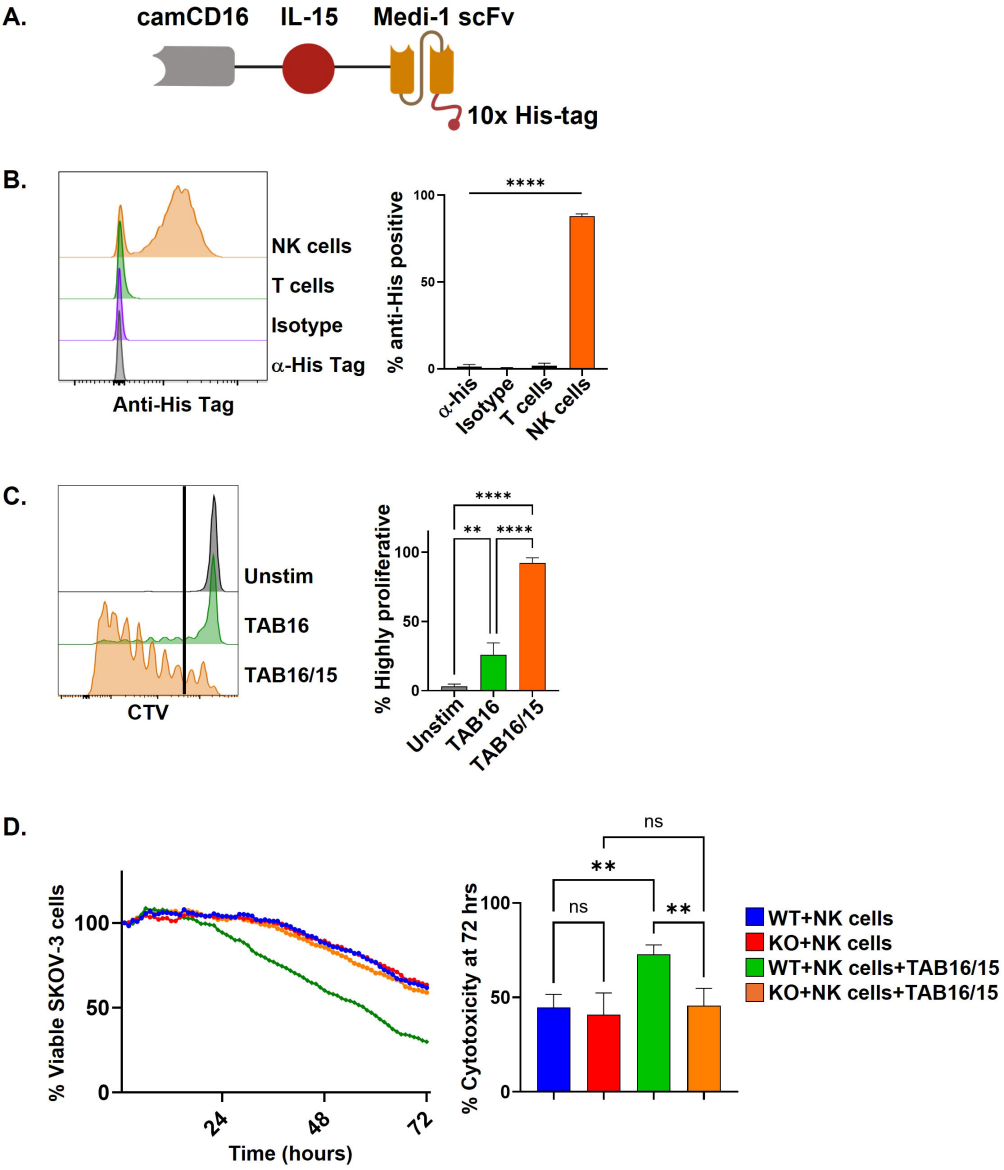
TAB16 modified with an IL-15 moiety (TAB16/15) maintains TAB16 function without additional IL-15. **(A)** Schematic of TAB16/15. **(B)** PBMCs were cultured with TAB16/15 (33 nM) for 30 minutes, washed thoroughly, and stained with anti-his tag antibody. Cell staining was analyzed by flow cytometry on T cells (CD56^–^ CD3^+^) and NK cells (CD56^+^ CD3^–^). Representative flow plot (left) and cumulative data (right). **(C)** PBMCs were labeled with CTV and cultured for 7 days with TAB16 (33nM) or TAB16/15 (33 nM). CTV dilution was analyzed by flow cytometry gating on NK cells (CD56^+^ CD3^–^). Representative flow plot (left), highly proliferated cells were gated at the indicated line, cumulative data graphed (right). **(D)** Enriched NK cells (>90%) were cultured with SKOV-3 NLR WT or SKOV-3 NLR KO cells with IL-15 (10 ng/ml) or TAB16/15 (8.25 nM). E:T ratio of 2:1. Cytotoxicity was assessed by IncuCyte live cell imaging. The percentage of SKOV-3 cells double normalized to target cells alone . Representative IncuCyte plot (left panel) and the percentage of cytotoxicity at 72 hours quantified with cumulative data (right panel). Mean +/- SD, n=3-4 donors. **p<0.01; ***p<0.001; ****p<0.0001; ns, not significant. Statistical significance was determined by one-way ANOVA with a Tukey *post hoc* test.

The use of TAB16 and IL-15 as separate components for research or clinical application is limited by lot variability and differing pharmacokinetic profiles. To address this, we generated a multi-engager complex consisting of Medi-1 scFv, cam16, and human IL-15, referred to as TAB16/15 (**Figure 7A**). This complex also contained a C-terminal 10x his tag. PBMCs were incubated with TAB16/15, washed, and then stained with an anti-his tag antibody. Similar to TAB16, TAB16/15 bound to NK cells and not T cells, maintaining NK cell specificity (**Figure 7B**). To assess function by the IL-15 moiety in TAB16/15, PBMCs were labeled with CTV and treated with TAB16 (33 nM) alone or TAB16/15 (33 nM) for 7 days. TAB16/15 induced significantly higher levels of NK cell proliferation than TAB16 alone (**Figure 7C**). We also performed an IL-15 bioassay (see Methods) to compare the functionality of TAB16/15 to IL-15. We found that TAB16/15 was ~10 times less potent than IL-15 by molarity (data not shown), indicating that Medi-scFv and/or camCD16 flanking the IL-15 moiety hindered its activity, as observed with other similarly designed multi-engager complexes (43, 45).

To assess the cytotoxic function of TAB16/15, enriched NK cells were co-cultured with SKOV-3 cells expressing or lacking ADAM17 at an E:T ratio of 2:1 and monitored by IncuCyte live cell imaging. NK cells demonstrated similar killing of SKOV-3 WT and KO cells in the presence of IL-15 (10 ng/ml) (**Figure 7D**). SKOV-3 KO cells were killed at an equivalent level by NK cells plus an equifunctional concentration of TAB16/15 (8.25 nM) as well (**Figure 7D**), indicating the IL-15 moiety sustained their cytolytic function. NK cells in the presence of TAB16/15, however, exhibited significantly increased cytotoxicity of SKOV-3 WT cells (**Figure 7D**), revealing a linkage between TAB16/15 and ADAM17 on SKOV-3 cells. The ovarian cancer cell lines OVCAR8 and PA-1 also express high levels of ADAM17 and HER2 (**Figure 5A**). Enriched NK cells were co-cultured with PA-1 or OVCAR8 cells at an E:T ratio of 2:1 in the presence of IL-15, IL-15 with trastuzumab, or TAB16/15 alone. TAB16/15 again significantly increased NK cell killing of the two tumor cell lines when compared to NK cells treated with IL-15 (**Figure 5B**), which was equivalent to NK cells in the presence of IL-15 and trastuzumab (**Figure 5B**). Taken together, our results establish TAB16/15 as a potent and selective NK cell engager that combines ADAM17 inhibition and tumor cell targeting with IL-15 stimulation, without the need for exogenous cytokine administration.

## Discussion

Harnessing NK cell cytotoxicity for cancer immunotherapy is a rapidly growing area of research (52). Antibody therapies that mediate ADCC offer a versatile and antigen-specific approach to tumor targeting (9, 53). To improve this process, multi-engager complexes are being developed that link NK cells to tumor antigens and contain a cytokine, such as IL-15, to further stimulate NK cells and modulate their anti-tumor effector functions (54–56). These approaches predominantly rely on the potent activating receptor CD16 for NK cell cytotoxicity. However, a key limitation of targeting CD16 is its rapid shedding from the NK cell surface by the protease ADAM17 (11–14). Approaches to prevent CD16 shedding have included engineering mutations in its cleavage region and mAbs directed at this site (11, 57, 58). In addition, we have previously reported that the mAb Medi-1 inhibits ADAM17 function and CD16 shedding, while also being engaged by CD16, thereby inducing cellular signaling that synergizes with IL-15 stimulation (30). However, Medi-1’s widespread reactivity, due to the ubiquitous expression of ADAM17 and its broad substrate profile, is possibly problematic (22). Additionally, CD16 polymorphisms affect Fc binding affinity and signaling (34, 35), potentially resulting in variability in CD16 signaling when engaging Medi-1.

To address these issues, we developed TAB16 containing a camelid VHH domain previously validated to bind CD16 with high affinity and uniformly across CD16 variants (54, 59). Unlike Medi-1, TAB16 demonstrates cell specificity by targeting CD16 and blocking ADAM17. Targeted inhibition of this proteolytic checkpoint positions TAB16 as a key component for NK cell engagers to prevent the shedding of vital receptors (e.g., CD16 and CD62L) induced by NK cell activation or stress, such as in the tumor microenvironment. Moreover, TAB16 enhanced NK cell activation and proliferation when used in combination with IL-15, even at reduced cytokine concentrations. This is particularly important given the dose-limiting toxicities associated with IL-15-based therapies (60, 61). Thus, by enhancing NK cell proliferation at reduced IL-15 concentrations, TAB16 could mitigate these toxicities while preserving therapeutic benefit.

The cam16 arm incorporated into TAB16 can also recognize CD16/FcγRIIIB (59), which is expressed by neutrophils (47). We observed that TAB16 bound to these cells and blocked ADAM17 activity. Additionally, approximately 10% of peripheral blood monocytes express CD16 (62, 63). Future studies will evaluate the effects of TAB16 on neutrophil anti-tumor effector functions and determine if TAB16 impacts CD16^+^ monocytes.

ADAM17 is overexpressed in various malignancies (23, 25–27), including ovarian cancer (24, 29). We demonstrate a dual functionality by TAB16 in that it can also link NK cells to ADAM17 when expressed at high levels by ovarian cancer cell lines and induce ADCC. TAB16, however, did not cause apparent NK cell fratricide upon linking CD16 and ADAM17 in a *cis* manner. This may be due to low ADAM17 levels in hematopoietic and healthy cells (64, 65). Considering that CD16 is expressed at high levels by resting NK cells (66), we speculate that attached TAB16 molecules exceed the available ADAM17 on the NK cell surface, resulting in an excess of available Medi-scFv that can engage ADAM17 when overexpressed by tumor cells (see **Graphical Abstract**). Moreover, the high levels of ADAM17 on tumor cells may increase TAB16 binding avidity at the lytic synapse and ADCC induction via CD16 signaling.

In addition to TAB16 linking NK cells to ADAM17 on certain tumor cells, this process may also result in ADAM17 inhibition in these cells. The proteolytic release of cell-surface EGFR ligands by ADAM17 is a regulatory step that triggers EGFR signaling, initiating downstream autocrine signaling and driving tumor progression (48). Additionally, MHC class I-related chain molecules A and B (MICA and MICB) and natural cytotoxicity triggering receptor 3 ligand 1 (NR3LG1 or B7-H6) are broadly expressed by tumor cells (67, 68), and are known substrates of ADAM17 (48, 69). MICA/B and NR3LG1 are ligands of the NK cell activating receptors NKG2D and NKp30, respectively, and blocking their shedding has been shown to increase tumor cell killing by NK cells (69, 70). Soluble forms of MICA/B can act as decoys, suppressing NK cell function by downregulating receptor expression. A focus of future studies is not only determining the effects of TAB16 on NK cell effector functions, but also investigating whether it impedes tumor-intrinsic signaling pathways that drive disease progression.

TAB16 is a modular platform that can be further modified to enhance NK cell effector functions. For instance, to augment NK cell activation and proliferation in the absence of exogenous IL-15, TAB16/15 incorporates IL-15 while retaining binding to CD16^+^ NK cells. TAB16/15 also mediates ADCC by binding to ADAM17 on tumor cells. It is possible to further modify TAB16/15 by incorporating a tumor-specific camelid VHH domain for dual tumor antigen targeting, thereby increasing therapeutic efficacy against heterogeneous tumors and reducing the risk of antigen escape. Our goal for future pre-clinical studies is to incorporate an additional tumor-targeting arm, optimized for ovarian cancer, into TAB16/15. A target of interest is B7-H3, which is highly expressed by epithelial ovarian cancer cells and stromal cells within the tumor microenvironment, and is a promising therapeutic target (45, 71–74). This adaptable platform lays the groundwork for additional studies aimed at optimizing NK cell-based immunotherapies across a range of solid tumors.

## Data Availability

The raw data supporting the conclusions of this article will be made available by the authors, without undue reservation.

## Supplementary material

Supplementary Figure 1 Verification of TAB16 and TAB16/15 expression. TAB16 (left) and TAB16/15 (right), analyzed by Western blot using an anti-his tag antibody after SDS-PAGE. The predicted molecular weight of TAB16 = 41.5 kDa and TAB16/15 = 55.9 kDa. The TAB16/15 showed a higher apparent molecular weight due to suspected posttranslational modifications.

Supplementary Figure 2 TAB16 selectivity for CD16^+^ leukocytes. **(A)** Flow cytometry of PBMCs cultured with 33 nM TAB16 for 30 minutes, thoroughly washed, and stained with an anti-his tag antibody along with antibodies to distinguish NK cells (CD56^+^ CD3^–^) and T cells (CD56^–^ CD3^+^). PBMCs treated with TAB16 were also stained with an isotype-matched negative control antibody (Isotype) to assess background staining levels. NK cells shown in red, T cells in black, NK cells stained with isotype control in blue. **(B)** Peripheral blood leukocyte populations are labeled (top). Whole leukocytes were incubated with TAB16 (33 nM) as described in A, neutrophils were gated based on FSC/SSC, and analyzed by flow cytometry for surface expression levels of CD16 and anti-his tag staining. **(C)** Whole leukocytes were activated with PMA (10 ng/ml) and ionomycin (1 μg/ml) for 30 minutes at 37°C in the presence of Medi-1 (33 nM) or TAB16 (33 nM). Neutrophils were gated based on FSC/SSC, and analyzed by flow cytometry for surface expression of CD62L. Data shown as mean +/– SD, n=3 donors. *p<0.05; **p<0.01; ****p<0.0001; ns, not significant. Statistical significance was determined by one-way ANOVA with a Tukey *post hoc* test or paired two-tailed Student’s t-test.

Supplementary Figure 3 NK cell and SKOV-3 co-culture live-cell imaging. Enriched NK cells were co-cultured with SKOV-3 WT NLR or SKOV-3 KO NLR cells at an E:T ratio of 2:1 for 72 hours in the presence of IL-15 (10 ng/ml), +/- TAB16 (33 nM). Representative images are shown at 24, 48, and 72 hours.

Supplementary Figure 4 TAB16 prevents CD16 cleavage during NK cell-mediated ADCC. Enriched NK cells were co-cultured with SKOV-3 WT cells at an E:T ratio of 1:1 for 5 hours in the presence of IL-15 (10 ng/ml), +/- TAB16 (33 nM), trastuzumab (5 μg/ml), or B7H3 BiKE (33nM). After extensive washing, cells were analyzed by flow cytometry for surface expression levels of CD16. Representative histogram (left) and cumulative data (right) shown. Data shown as mean +/– SD, n=4 donors. ****p<0.0001; ns = not significant. Statistical significance was determined by one-way ANOVA with a Tukey *post hoc* test.
