# Supplementary figures and images for: Dual-functioning Targeted ADAM17 Blocker CD16 (TAB16) mediates selective ADAM17 inhibition in NK cells and engages overexpressed ADAM17 in tumor cells to induce cytotoxicity
Source: Front Immunol. 2026 Jan 30;17:1714022. doi: 10.3389/fimmu.2026.1714022 (PMC12901421; doi:10.3389/fimmu.2026.1714022)

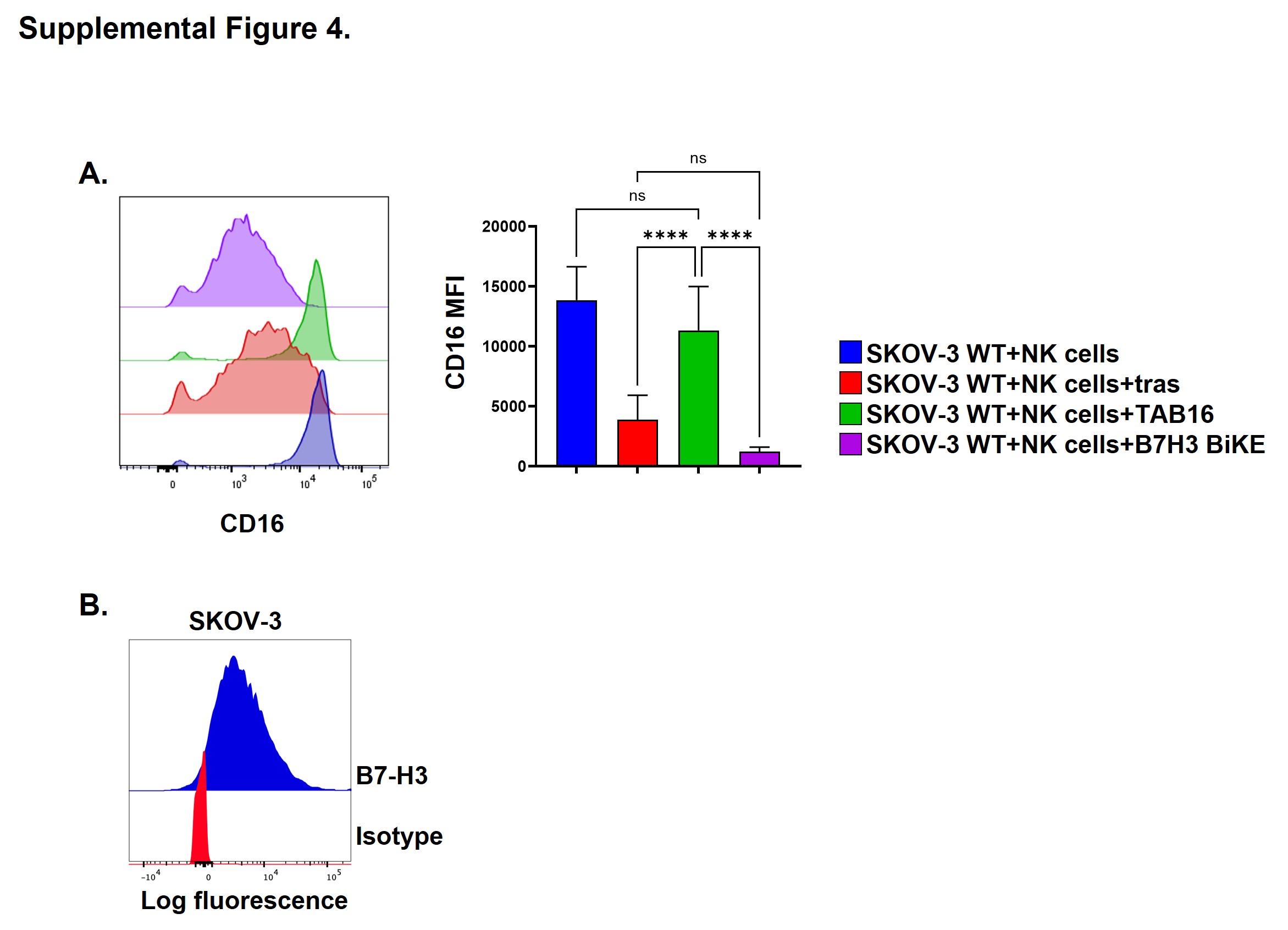

Supplement: Supplementary Figure 1 — Verification of TAB16 and TAB16/15 expression. TAB16 (left) and TAB16/15 (right), analyzed by Western blot using an anti-his tag antibody after SDS-PAGE. The predicted molecular weight of TAB16 = 41.5 kDa and TAB16/15 = 55.9 kDa. The TAB16/15 showed a higher apparent molecular weight due to suspected posttranslational modifications. [file Supplementaryfile1.zip › Supplementary Figure 4.JPEG]

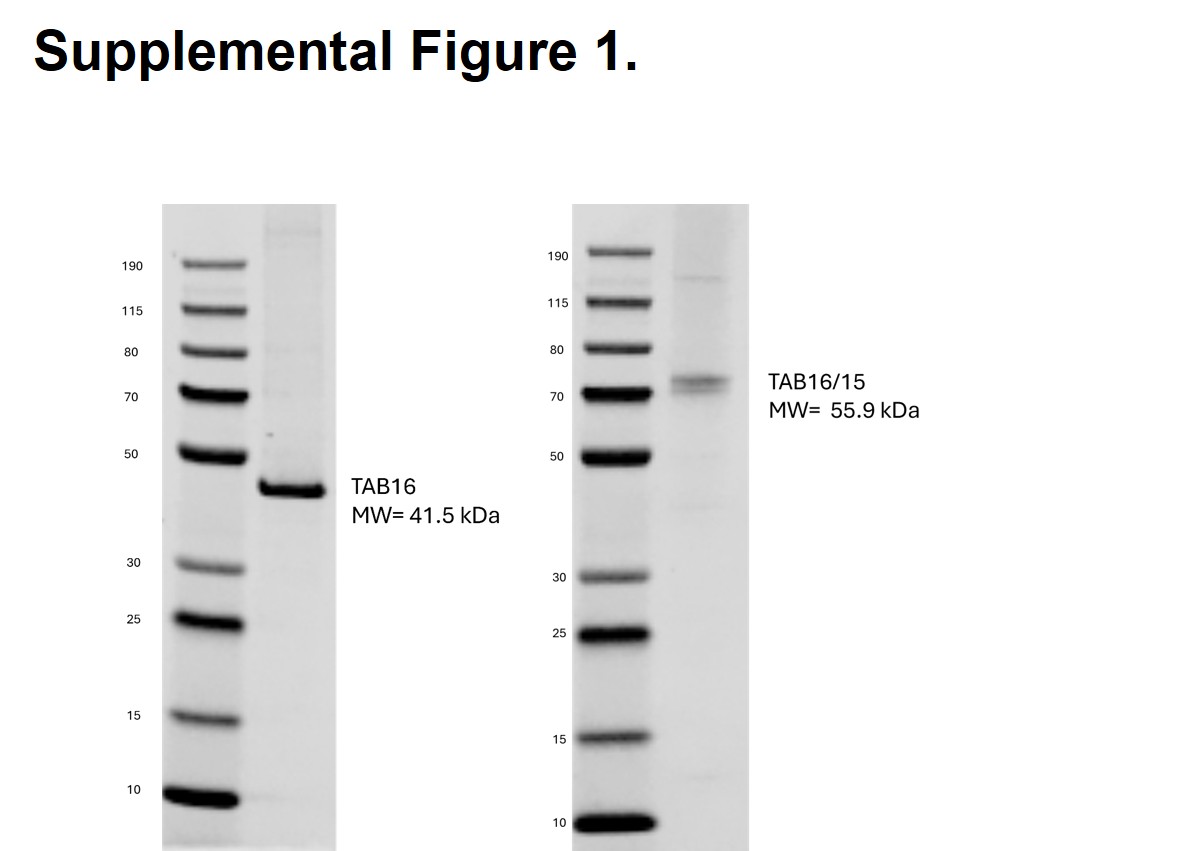

Supplement: Supplementary Figure 1 — Verification of TAB16 and TAB16/15 expression. TAB16 (left) and TAB16/15 (right), analyzed by Western blot using an anti-his tag antibody after SDS-PAGE. The predicted molecular weight of TAB16 = 41.5 kDa and TAB16/15 = 55.9 kDa. The TAB16/15 showed a higher apparent molecular weight due to suspected posttranslational modifications. [file Supplementaryfile1.zip › Supplementary Figure 1.JPEG]

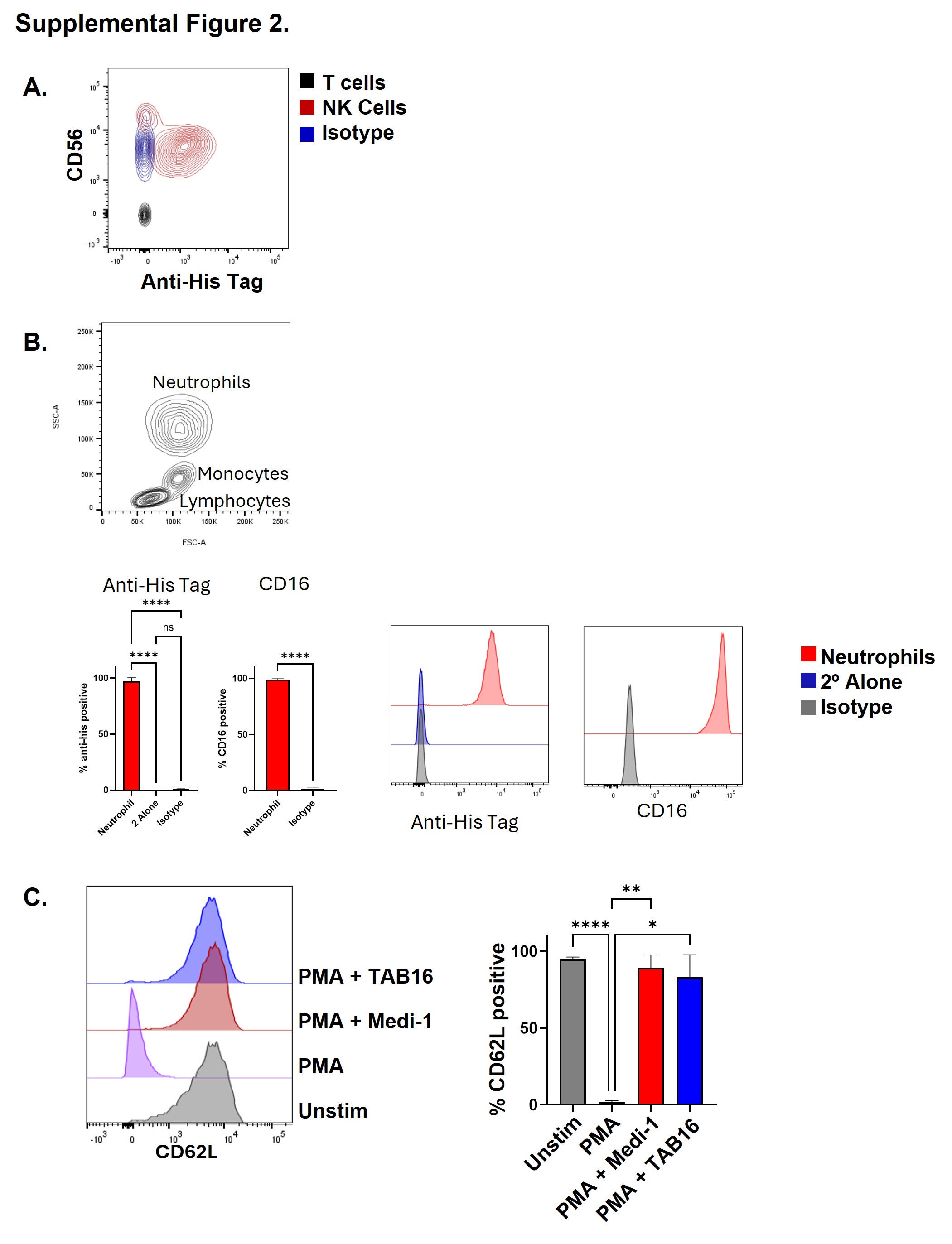

Supplement: Supplementary Figure 1 — Verification of TAB16 and TAB16/15 expression. TAB16 (left) and TAB16/15 (right), analyzed by Western blot using an anti-his tag antibody after SDS-PAGE. The predicted molecular weight of TAB16 = 41.5 kDa and TAB16/15 = 55.9 kDa. The TAB16/15 showed a higher apparent molecular weight due to suspected posttranslational modifications. [file Supplementaryfile1.zip › Supplementary Figure 2.JPEG]

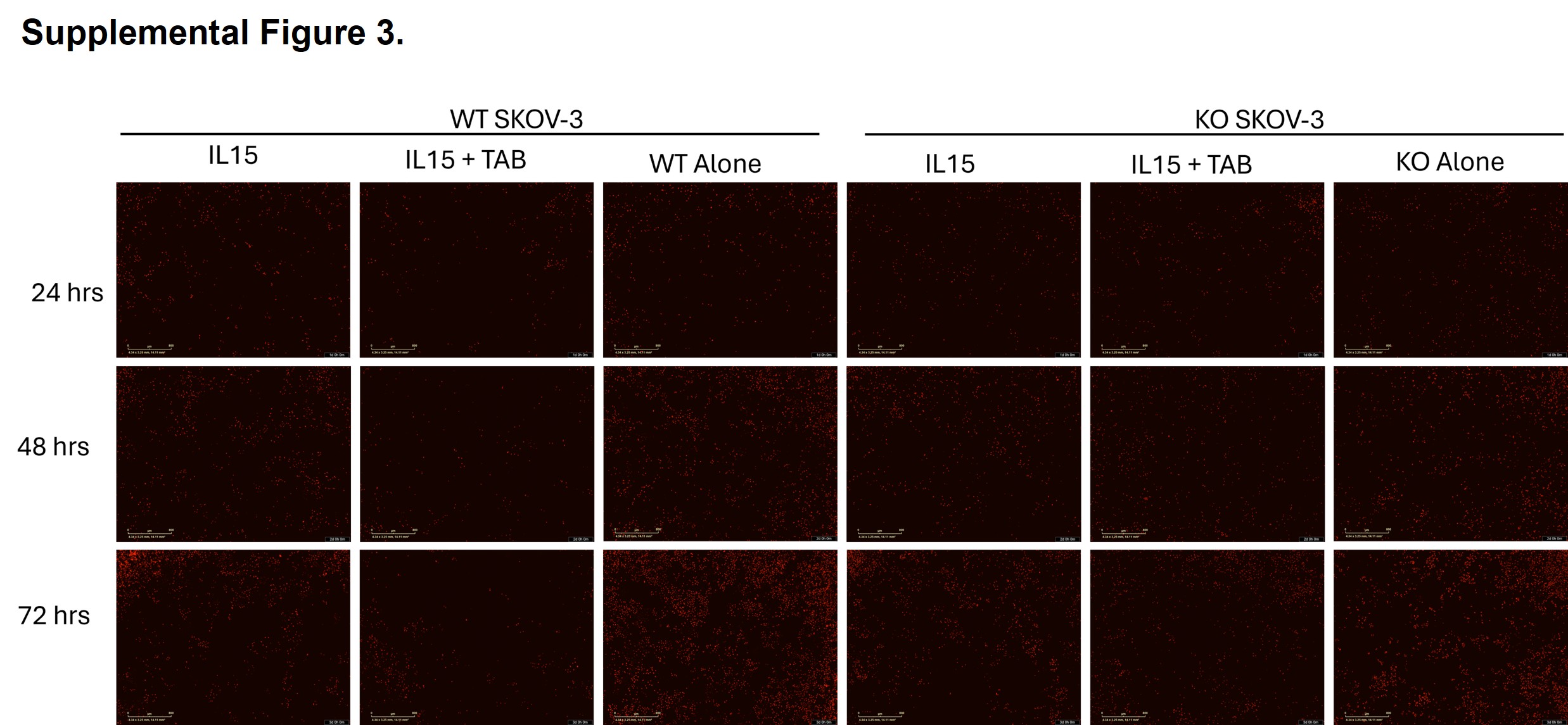

Supplement: Supplementary Figure 1 — Verification of TAB16 and TAB16/15 expression. TAB16 (left) and TAB16/15 (right), analyzed by Western blot using an anti-his tag antibody after SDS-PAGE. The predicted molecular weight of TAB16 = 41.5 kDa and TAB16/15 = 55.9 kDa. The TAB16/15 showed a higher apparent molecular weight due to suspected posttranslational modifications. [file Supplementaryfile1.zip › Supplementary Figure 3.JPEG]
